# Association of Orthostatic Hypotension With Cerebral Atrophy in Patients With Lewy Body Disorders

**DOI:** 10.1212/WNL.0000000000012342

**Published:** 2021-08-24

**Authors:** Andrea Pilotto, Alberto Romagnolo, Andrea Scalvini, Mario Masellis, Yasushi Shimo, Laura Bonanni, Richard Camicioli, Lily L. Wang, Alok K. Dwivedi, Katherine Longardner, Federico Rodriguez-Porcel, Mark DiFrancesco, Joaquin A. Vizcarra, Elisa Montanaro, Simona Maule, Alessandro Lupini, Carmen Ojeda-López, Sandra E. Black, Stefano Delli Pizzi, Myrlene Gee, Ryota Tanaka, Kazuo Yamashiro, Taku Hatano, Barbara Borroni, Roberto Gasparotti, Maria C. Rizzetti, Nobutaka Hattori, Leonardo Lopiano, Irene Litvan, Alberto J. Espay, Alessandro Padovani, Aristide Merola

**Affiliations:** From the Neurology Unit (A. Pilotto, A.S., B.B., A.L., A. Padovani), Department of Clinical and Experimental Sciences, and Neuroradiology Unit (R.G.), Department of Medical and Surgical Specialties, Radiological Sciences and Public Health, University of Brescia; Parkinson's Disease Rehabilitation Centre (A. Pilotto, M.C.R.), FERB ONLUS–S. Isidoro Hospital, Trescore Balneario, Bergamo; Department of Neuroscience “Rita Levi Montalcini” (A.R., E.M., L.L.) and Autonomic Unit (S.M.), Department of Medical Sciences, University of Turin, Italy; Department of Medicine (Neurology) (M.M., C.O.-L., S.E.B.), University of Toronto; Hurvitz Brain Sciences Program (M.M., C.O.-L., S.E.B.), Sunnybrook Research Institute, Sunnybrook Health Sciences Centre, Toronto, Ontario, Canada; Department of Neurology (Y.S., R.T., K.Y., T.H., N.H.), Juntendo University Graduate School of Medicine, Tokyo, Japan; Department of Neuroscience Imaging and Clinical Sciences (L.B., S.D.P.), University G. d’Annunzio of Chieti-Pescara, Chieti, Italy; Department of Medicine and Neuroscience and Mental Health Institute (R.C., M.G.), University of Alberta, Edmonton, Canada; Department of Radiology (L.L.W.), and Gardner Family Center for Parkinson's Disease and Movement Disorders (A.J.E.), Department of Neurology, University of Cincinnati, OH; Department of Molecular and Translational Medicine (A.K.D.), Texas Tech University Health Sciences Center, El Paso; Parkinson and Other Movement Disorders Center (K.L., I.L.), Department of Neurosciences, University of California, San Diego, La Jolla; Department of Neurology (F.R.-P.), Medical University of South Carolina, Charleston; Imaging Research Center (M.D), Department of Radiology, Cincinnati Children's Hospital Medical Center; University of Cincinnati College of Medicine (M.D.), OH; Department of Neurology (J.A.V.), Emory University, Atlanta, GA; ASST Spedali Civili Hospital (R.G.), Brescia, Italy; and Department of Neurology (A.M.), The Ohio State University, Columbus .

## Abstract

**Objective:**

To evaluate whether orthostatic hypotension (OH) or supine hypertension (SH) is associated with brain atrophy and white matter hyperintensities (WMH), we analyzed clinical and radiologic data from a large multicenter consortium of patients with Parkinson disease (PD) and dementia with Lewy bodies (DLB).

**Methods:**

Supine and orthostatic blood pressure (BP) and structural MRI data were extracted from patients with PD and DLB evaluated at 8 tertiary-referral centers in the United States, Canada, Italy, and Japan. OH was defined as a systolic/diastolic BP fall ≥20/10 mm Hg within 3 minutes of standing from the supine position (severe ≥30/15 mm Hg) and SH as a BP ≥140/90 mm Hg with normal sitting BP. Diagnosis-, age-, sex-, and disease duration–adjusted differences in global and regional cerebral atrophy and WMH were appraised with validated semiquantitative rating scales.

**Results:**

A total of 384 patients (310 with PD, 74 with DLB) met eligibility criteria, of whom 44.3% (n = 170) had OH, including 24.7% (n = 42) with severe OH and 41.7% (n = 71) with SH. OH was associated with global brain atrophy (*p* = 0.004) and regional atrophy involving the anterior-temporal (*p* = 0.001) and mediotemporal (*p* = 0.001) regions, greater in severe vs nonsevere OH (*p* = 0.001). The WMH burden was similar in those with and without OH (*p* = 0.49). SH was not associated with brain atrophy (*p* = 0.59) or WMH (*p* = 0.72).

**Conclusions:**

OH, but not SH, was associated with cerebral atrophy in Lewy body disorders, with prominent temporal region involvement. Neither OH nor SH was associated with WMH.

Orthostatic hypotension (OH), defined as blood pressure (BP) drop ≥20/10 mm Hg (systolic/diastolic) within 3 minutes of standing,^1^ and supine hypertension (SH), defined as supine BP ≥140/90 mm Hg with normal sitting BP,^2^ are hemodynamic manifestations of cardiovascular dysautonomia, commonly associated with Lewy body disorders. It has been estimated that 30% of patients with Parkinson disease (PD) and 30% to 70% with dementia with Lewy bodies (DLB) are affected by OH and that ≈40% to 70% of cases of OH are complicated by SH.^3^

Multiple studies have documented an association between OH and cognitive impairment, suggesting that common pathogenic mechanisms might be involved in cognitive and autonomic dysfunction or that recurrent episodes of cerebral hypoperfusion and hyperperfusion might negatively affect the cognitive function of patients with Lewy body disorders.^3-7^ These hypotheses are supported by small imaging studies showing regional brain atrophy in the insula^8^ and the cholinergic pathways^5^ and by the assumption that hemodynamic dysfunction might result in transient cognitive impairment or chronic cerebrovascular damage reflected by white matter hyperintensities (WMH).^1,9,10^

Using a large multicenter repository of imaging and clinical data, we sought to analyze the association of OH and SH with global and regional brain atrophy and with WMH.

## Methods

We searched the clinical and imaging repositories of a large multicenter consortium constituted by 8 specialized Movement Disorder and Dementia Centers in the United States (University of Cincinnati), Canada (University of Toronto, University of Alberta), Italy (University of Brescia, University of Torino, University of Chieti-Pescara, Parkinson's Disease Rehabilitation Centre Trescore Balneario), and Japan (Juntendo University, Tokyo).

### Inclusion and Exclusion Criteria

Patients with PD and DLB meeting all of the inclusion and none of the exclusion criteria listed below were enrolled in the study. Inclusion criteria were (1) clinical diagnosis of idiopathic PD as per the Movement Disorders Society (MDS) criteria^11^ or DLB as per the International DLB Consortium criteria^12^; (2) standardized orthostatic BP assessment (patient lying supine for at least 5 minutes and then standing for 3 minutes); (3) stable dosage of dopaminergic and vasopressor medications for at least 4 weeks before the orthostatic BP assessment; (4) brain MRI, including T1-weighted and T2-weighted sequences acquired at ≥1.5T; (5) availability of MDS–Unified Parkinson's Disease Rating Scale (MDS-UPDRS) section III (motor symptoms)^13^ or UPDRS score at the time of BP assessment; and (6) availability of Montreal Cognitive Assessment (MoCA)^14^ or Mini-Mental State Examination (MMSE)^15^ scores at the time of BP assessment.

Exclusion criteria were (1) nonneurogenic OH, defined as Δheart rate/Δsystolic BP ratio ≥0.5 bpm/mm Hg^16^; (2) comorbid diabetic neuropathy or other disorders associated with deficits within the autonomic nervous system^17^; (3) nonneurogenic OH due to treatment with antihypertensive drugs or any therapy with an effect on BP such as α-adrenergic antagonists for prostate disorders; (4) clinical history of acute cerebrovascular disease (ischemic/hemorrhagic stroke and/or TIA); (5) other neurologic disorders or medical conditions potentially associated with cognitive deficits, including kidney and liver metabolic diseases^18^; (6) any atypical clinical features lowering the diagnostic certainty of PD or DLB; (7) major psychiatric diseases requiring long-term use of typical antipsychotic medications; and (8) history of drug or alcohol abuse.

### Definition of OH and SH

BP and heart rate were evaluated in the sitting, supine (after at least 5 minutes of rest), and standing positions. OH was defined as a BP fall ≥20 mm Hg systolic or 10 mm Hg diastolic within 3 minutes of standing^19^ from the supine position and rated as severe OH if the BP fall was ≥30 mm Hg systolic or 15 mm Hg diastolic BP.^20^ SH was defined as supine systolic BP ≥140 mm Hg or diastolic BP ≥90 mm Hg; severe SH was defined as supine systolic BP values of ≥180 mm Hg or diastolic BP values of ≥110 mm Hg in patients with normal sitting BP.^2^

### Imaging Data

T1-weighted and T2-weighted images were exported in a Digital Imaging and Communications in Medicine format and analyzed in a centralized fashion by 4 independent raters as detailed in the statistical methods.

Brain atrophy was evaluated in 6 distinct regions (anterior-cingulate; orbitofrontal; anterior-temporal; fronto-insular; mediotemporal; posterior) on T1-weighted images according to the semiquantitative approach described in the work of Harper and colleagues^21^ and rated as follows: 0 = closed sulcus; 1 = sulcal opening; 2 = sulcal widening; 3 = severe sulcal widening with volume loss; and 4 = profound volume loss (score 4 applicable only for medial and anterior temporal lobe atrophy).

WMH were assessed in 4 distinct regions (periventricular white matter; deep white matter; basal ganglia plus internal capsule; and infratentorial white matter) on T2-weighted images and rated, according to Scheltens et al.^22^ as follows: 0 = no white matter lesion; 1 = punctiform white matter lesions; 2 = early confluent white matter lesions; and 3 = confluent white matter lesions. The final analyses were performed by adding the separate scores recorded for regions in the left and right hemispheres, resulting in scores between 0 and 6 (0–8 for temporal atrophy).

On the basis of the work by Harper et al.,^21^ the interrater reliability of scales of atrophy ranged from 0.5 to 0.79 for different regions with average rater scores for all scales (≥0.73).^22^ A random sample of 36 MRIs were preliminarily evaluated by the 4 raters to estimate the intraclass correlation coefficient, which was deemed acceptable if >0.70 (e-table 1, data available from Dryad, doi.org/10.5061/dryad.6q573n5zd).

### Clinical Data

The medical records were searched for the following demographic/clinical information within a time frame of 3 months from MRI: sex, age, age at disease onset, ethnicity, family history of neurologic or psychiatric disorders, diabetic neuropathy, hypertension, hypercholesterolemia, history of hemorrhagic/ischemic stroke or TIA, myocardial infarction, coronary artery bypass graft, angioplasty or stenting, atrial fibrillation, and valvulopathy.^18^ The MDS-UPDRS-III or UPDRS-III score, Hoehn & Yahr stage, and MoCA or MMSE score were also collected. A conversion from MMSE to MoCA score was applied as needed with the Lawton et al.^23^ formula, and the MoCA total score was used as a measure of global cognition. A conversion from UPDRS-III to MDS-UPDRS-III score was applied using the formula developed by Goetz and coauthors^24^ when needed.

Dopaminergic therapies, including levodopa, dopamine agonists, monoamine-oxidase-B inhibitors, and catechol-O-methyltransferase inhibitors, were recorded and used to calculate the total levodopa equivalent daily dose as per the conversion table proposed by Tomlinson et al.^25^ The use of medications for diabetes, hypertension, hyperlipidemia, depression, and psychosis was also recorded.

### Sample Size Calculation

Applying the adjusted difference of 0.53 units of atrophy (95% confidence interval [CI] 0.05–1.02) in individuals with and without OH in WMH (15.6 ± 9.6 vs 11 ± 8.2 for total score) reported in previous studies^26,27^ and assuming an equal variance between groups indicated that a sample size of at least 90 OH+ and 90 OH− individuals (total = 180) was estimated to achieve 80% power for WMH assessment with 1% level of significance using a multiple linear regression analysis. The combined coefficient of covariation *R*^2^ was assumed to be 20% with covariates. The level of significance was adjusted to 1% due to multiple comparisons. Assuming a prevalence of OH of 40% (95% CI 23%–38%) in Lewy body disorders with similar effect sizes as considered for WMH, it was estimated that 350 cases would be needed to have >80% power to evaluate the effects of OH groups after adjustment for diagnosis (PD vs DLB) on MRI using multiple linear regression analysis. The sample size was explored for different OH prevalence scenarios (30%–60%) using Power Analysis and Sample Size Software (PASS 14 Power Analysis and Sample Size Software, 2015, NCSS, LLC, Kaysville, UT; ncss.com/software/pass).

### Statistical Analyses

Demographic variables, clinical characteristics, and vascular risk factors were compared in patients with and without OH (subdivided further into OH and severe OH) using analysis of variance/multiple linear analysis, with study group as main factor, and the χ^2^ test for continuous and dichotomous variables, respectively. Quantitative data were presented as mean ± SD. Analysis of covariance was used to estimate differences in semiquantitative scales for the assessment of regional cerebral atrophy and WMH (dependent variables) between the 3 OH groups (without OH, OH, and severe OH—independent variables) with adjustment for diagnosis (PD vs DLB), age, sex, years of education, and disease duration (covariates). The effect size (mean difference and 95% CI) of OH groups on each region of cerebral atrophy and WMH was determined with multiple linear regression analysis. In addition, the Cohen effect size was estimated for each outcome in relation to OH groups with multiple ordinary linear regression analysis using STATA 15.1 codes.

The same analysis using *t* test and χ^2^ test for demographics and analysis of covariance for atrophy and WMH rating was performed with SH as an independent variable in the group of patients with OH only. Multiple-comparison adjustment with Bonferroni correction was applied to the significance level (α) for single atrophy regions (α = 0.05/6 = 0.008) and WMH (α = 0.05/5 = 0.01).

Analysis of covariance assumption of homogeneity of regression slopes was verified. Statistical tests were performed with Statistical Package for the Social Sciences (SPSS 21.0 for Macintosh, Chicago, IL). The 2-tailed significance threshold was set at 0.016 in post hoc analyses of within-group comparisons.

### Standard Protocol Approvals, Registrations, and Patient Consents

This study received Institutional Review Board/ethics committee approval at all participating centers and was conducted in accordance with Good Clinical Practice and any applicable national and local regulations. The General Data Protection Regulation requirements for data collection were met. Written informed consent was obtained from all participants.

### Data Availability

The data that support the findings of this study are available from the corresponding author on reasonable request.

## Results

### Patients

**Figure 1:**
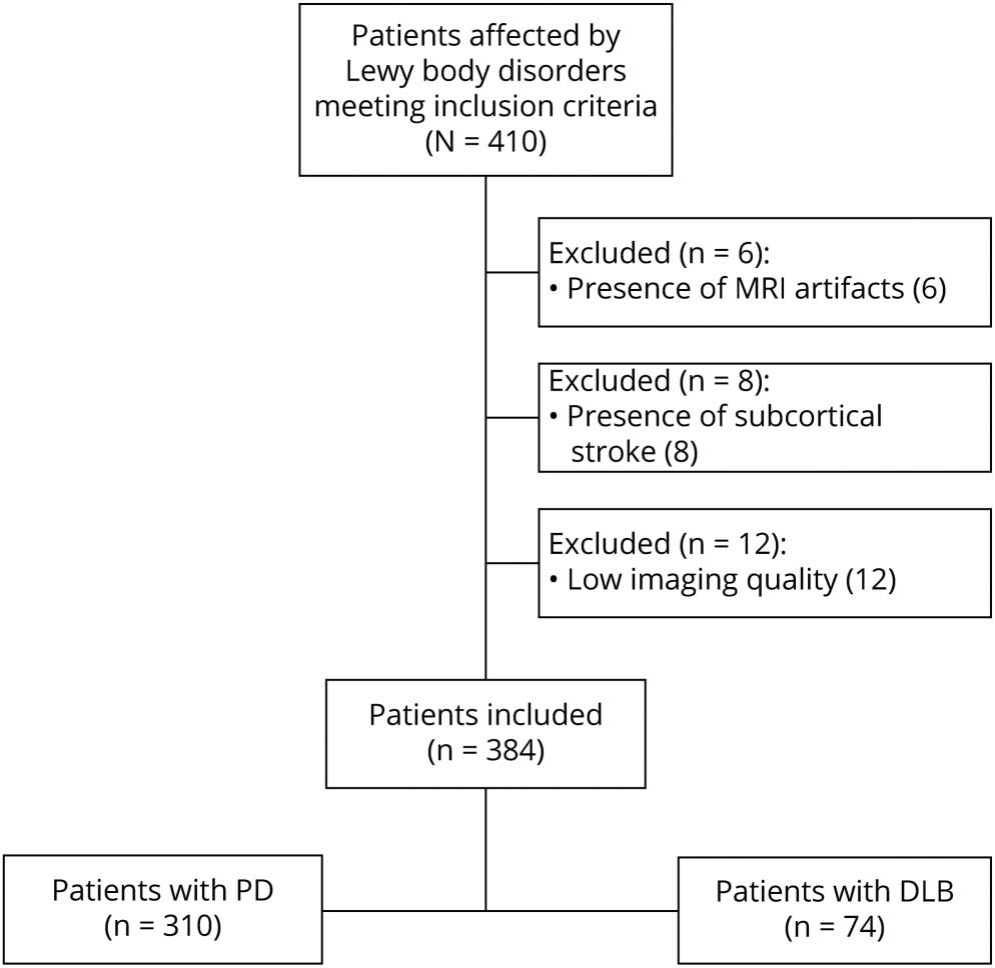
Study Flowchart DLB = dementia with Lewy bodies; PD = Parkinson disease.

A total of 410 patients were initially included in the study. Of these, 6 were excluded due to MRI motion artifacts, 8 due to subcortical ischemic strokes (4 without OH, 3 with OH, and 1 with severe OH), and 12 due to low imaging quality insufficient for accurate brain atrophy rating (figure 1). Of the remaining 384 patients (310 with PD and 74 with DLB), 44.3% (n = 170) had OH. Among patients with OH, 24.7% (n = 42) had severe OH and 41.7% had SH (n = 71). Patients with PD were younger (65.8 ± 10.3 years vs 79.1 ± 7.2 years) and had longer disease duration (9.2 ± 5.3 years vs 6.6 ± 4.5 years) and better cognitive scores (MoCA score 24.3 ± 2.9 vs 16.1 ± 5.1) than those with DLB. No differences were observed in the OH distribution between PD and DLB (table 1).

**Table 1:**
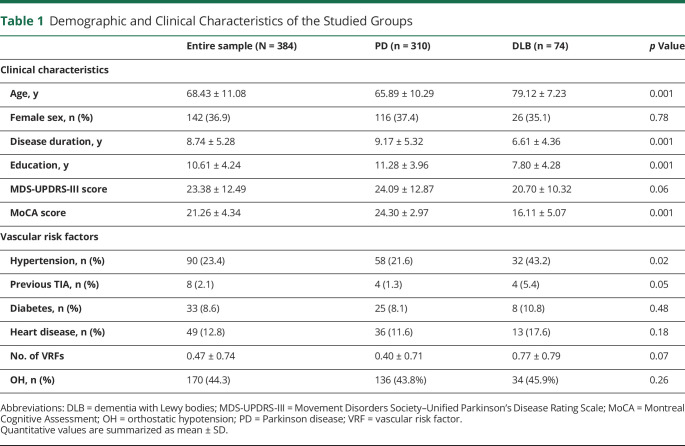
Demographic and Clinical Characteristics of the Studied Groups

Patients with OH had longer disease duration (*p* = 0.02) and higher MDS-UPDRS-III scores (*p* = 0.02) compared to patients without OH, with no differences in age, sex distribution, and vascular risk factors (table 2). Patients with SH had more vascular risk factors (hypertension, diabetes, dyslipidemia, cardiovascular disease) but similar age, sex distribution, disease duration, motor performance, and cognitive impairment compared to those without SH (table 2).

**Table 2:**
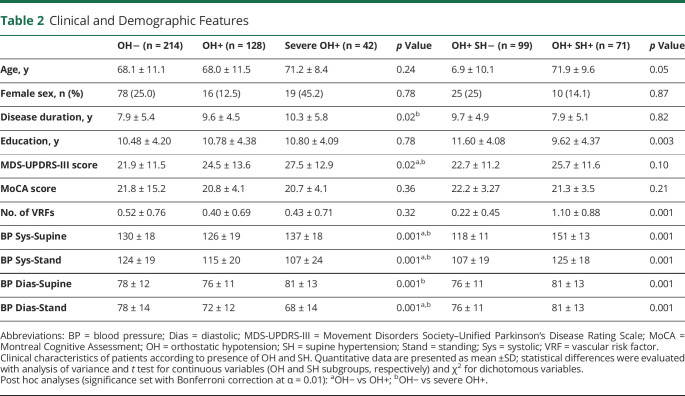
Clinical and Demographic Features

### OH-Associated Imaging Data

**Figure 2:**
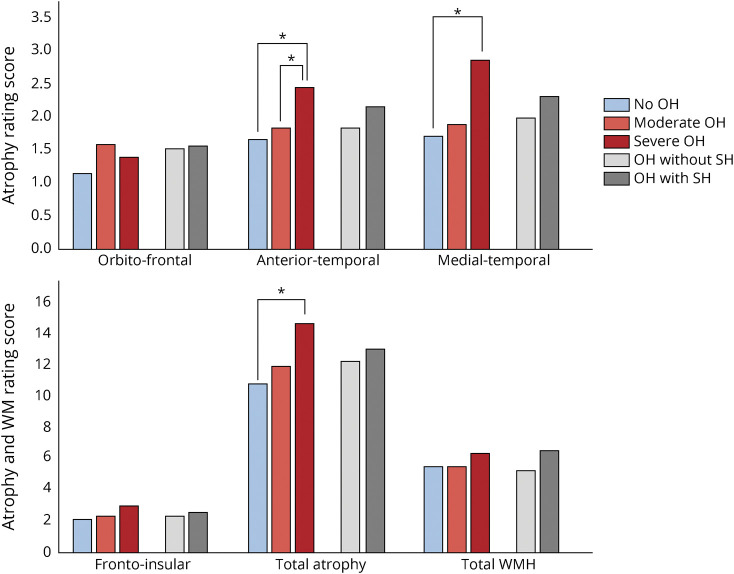
Atrophy and WMH Rating According to OH and SH Subgroups OH = orthostatic hypotension; SH = supine hypertension; WM = white matter; WMH = white matter hyperintensities. Post hoc analyses (*significance set with Bonferroni correction at α = 0.016).

Age-, sex-, diagnosis-, education-, and disease duration–adjusted data showed an association of OH with both global cerebral atrophy (*p* = 0.004) and regional atrophy involving the anterior-temporal (*p* = 0.001) and mediotemporal (*p* = 0.001) regions (table 3 and figure 2). Post hoc analyses showed greater global atrophy in patients with severe OH vs patients without OH (*p* = 0.006); patients with severe OH showed greater anterior temporal atrophy compared to both patients with OH (*p* < 0.001) and patients without OH (*p* < 0.001) and greater medial temporal atrophy compared to patients without OH (*p* = 0.002) (table 4). No differences were observed in the global and regional scoring of WMH between patients with OH and those without OH (table 3 and figure 2).

**Table 3:**
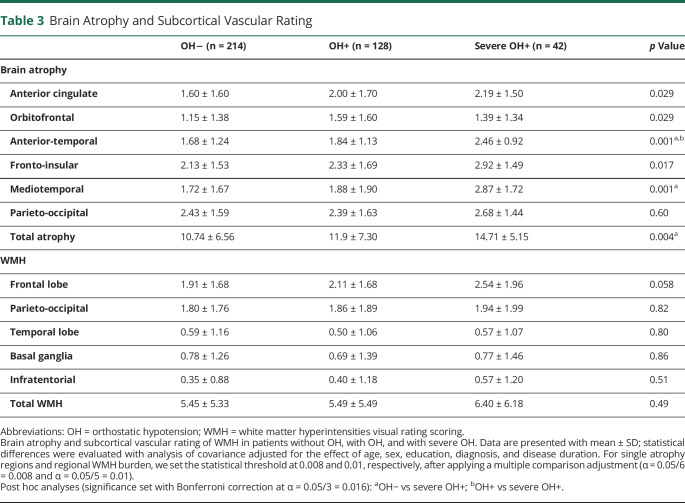
Brain Atrophy and Subcortical Vascular Rating

**Table 4:**
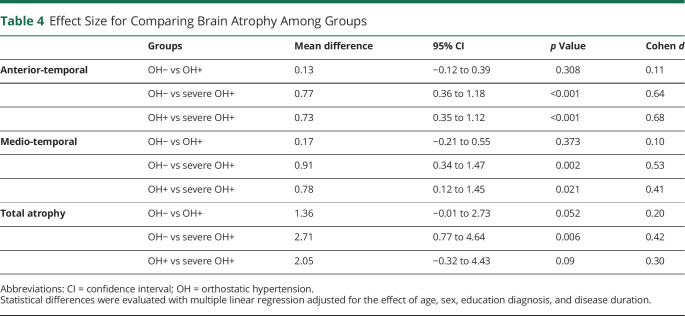
Effect Size for Comparing Brain Atrophy Among Groups

### SH-Associated Imaging Data

Age-, sex-, diagnosis-, education-, and disease duration–adjusted data showed no associations between SH or severe SH and global cerebral atrophy (*p* = 0.59 and *p* = 0.74, respectively), regional atrophy (*p* ≥ 0.07), or WMH (*p* ≥ 0.57) (table 5).

**Table 5:**
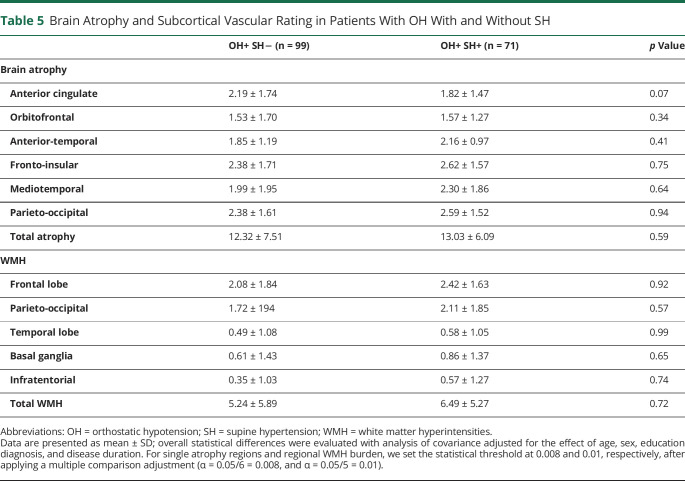
Brain Atrophy and Subcortical Vascular Rating in Patients With OH With and Without SH

## Discussion

Clinical and neuroimaging data from 384 patients with Lewy body disorders demonstrated that OH is associated with global and regional brain atrophy involving the anterior-temporal and mediotemporal regions, more pronounced in those with severe OH. No differences in WMH burden were detected in patients with and without OH or SH. In addition, SH was not associated with global or regional brain atrophy.

A growing number of studies have reported OH as one of the strongest predictors of cognitive outcomes in PD and DLB.^3,28^ Small single-center studies documented increased α-synuclein cortical and subcortical pathology in patients with OH,^29^ suggesting the association with a malignant disease phenotype, potentially worsened by acute and chronic cerebral hypoperfusion.^4,9^ Others proposed that the hemodynamic stress due to OH and SH might cause chronic damage to the small brain vessels, resulting in WMH, which can contribute to dementia in Lewy body disorders.^10,30^ To date, however, no studies have adequately addressed the impact of OH and SH on brain structural changes.

Whether repetitive hypotensive episodes contribute to these adverse outcomes through direct hypoxic damage of vulnerable areas or are merely associated with a more aggressive clinical subtype of Lewy body pathology remains unclear. The possibility exists that chronic hypoxia might trigger or accelerate the progression of neurodegenerative mechanisms. Experimental studies from aging animals showed that chronic brain hypoperfusion yields synaptic changes, metabolic dysregulation, cholinergic receptor loss, protein synthesis abnormalities, and visuospatial deficits.^31,32^ In addition, aging animals kept for prolonged periods of time after chronic brain hypoperfusion showed a tendency to develop neuronal damage in the hippocampal region and temporoparietal cortex.^33^ In a rat model of Alzheimer disease, chronic hypoxia was associated with increased deposition of β-amyloid in the frontal cortex and hippocampus and hyperphosphorylated tau in the temporal cortex.^34^ Overall, these findings support the hypothesis that chronic hypoxia might interfere with the cellular metabolic pathways already impaired by the ongoing neurodegenerative processes, ultimately leading to a faster progression of the neurodegenerative damage. However, the extent to which these pathogenic mechanisms apply to PD and DLB remains to be clarified.

The results of our study, adjusted for age, sex, disease duration, education, and vascular comorbid conditions, showed that OH is independently associated with global brain atrophy, more prominently in the temporal regions. The involvement of the anterior-temporal and mediotemporal lobes is critical because these regions have been directly associated with the progression of dementia in Lewy body disorders.^35,36^ We also found that OH has no effect on subcortical WMH burden. This finding clarifies a highly controversial point in the literature. A study of 44 patients with PD evaluated with cardiovascular autonomic testing and brain imaging found a similar WMH burden in patients with and without OH, suggesting that OH-associated cognitive deficits could not be explained by subcortical vascular disease.^37^ However, 3 other studies based on simple bedside BP measurements yielded opposite results.^27,38,39^ These conflicting findings might be related partly to the inclusion of patients with nonneurogenic OH, wherein there may be a greater role for vascular risk factors.^18^ In this study, we included only patients with neurogenic OH and stratified for OH severity and concomitant presence of SH to analyze subcategories of patients at potentially higher risk of microvascular damage. We found that neither OH nor SH was associated with a significantly higher burden of WMH, which can be explained by the fact that WMH require years of chronic vascular shear stress, whereas OH and SH are paroxysmal by definition, with acute episodic complications such as falls^20,40,41^ and cognitive fluctuations.^42^

Taking advantage of our large dataset, we also explored the impact of SH, which was not possible in prior smaller cohorts. Data from patients with chronic essential hypertension suggest that SH increases the risk of cardiovascular comorbid conditions,^43^ and a recent study found an association between SH and multiorgan damage in patients with pure autonomic failure, those with multiple system atrophy, and some cases of PD.^44^ However, in our analysis of 170 patients with Lewy body disorders with OH, 71 of whom had concomitant SH, we did not find an association between SH and brain atrophy or subcortical WMH burden. While we cannot exclude that a long-term follow-up analysis of patients with SH might reveal signs of cerebrovascular organ damage, our findings suggest that SH may have a smaller impact on brain parenchyma than essential hypertension, possibly because of its paroxysmal rather than chronic nature.^43^ This outcome can inform therapeutic protocols for the management of hemodynamic autonomic dysfunction in patients with PD and DLB because the successful treatment of OH often requires accepting a higher frequency of SH. Our data seem to suggest that this can be achieved with minimal impact on the vulnerable cortical and subcortical structures.

Several limitations should be acknowledged. First, we used semiquantitative scales for the assessment of brain atrophy. Despite extensive validation, these scales remain less sensitive than voxel-based morphometry analyses or fully quantitative region-of-interest analyses, especially for the posterior cortical regions. However, this would not be feasible for a retrospective study because most clinical brain MRIs do not include a volumetric T1 sequence for such purpose. A systematic and prospective acquisition of clinical, hemodynamic, and imaging data has already been initiated in selected centers and will be critical to confirm these results. Similarly, the collection of biological samples such as CSF will allow the evaluation of biomarkers, which may identify the underlying pathologic processes associated with the observed neuroimaging findings and evaluate the relationship with Alzheimer disease copathology.^45^ Second, our observational study design is inevitably prone to selection biases, which might have played a role in the observed outcomes. It is possible that the inclusion of patients with available standardized BP assessments in the supine and standing position may have introduced a bias toward the selection of those reporting orthostatic symptoms. In fact, the OH prevalence observed in our study (44%) is slightly higher than the average reported in the literature (≈30%).^46^ Third, the lack of extensive cognitive assessments limited our analyses to measures of global cognition. More comprehensive cognitive testing and prospective follow-up assessments are required to evaluate the impact of OH/SH on specific neuropsychological deficits. Fourth, the cardiovascular autonomic assessment was limited to the study of BP and heart rate. A more extensive battery of cardiovagal, adrenergic, and sudomotor testing will allow distinguishing pathogenic mechanisms involving different components of the autonomic nervous system. Finally, the lack of longitudinal assessments precluded the possibility of studying the effect of vasopressor treatments on the rate of brain atrophy progression.^47^ Clarifying this point will be critical to ascertain the extent to which brain atrophy represents a consequence rather than a cause of OH, a question of critical importance to inform the development of therapeutic protocols for the management of OH and SH.

Despite the limitations associated with an observational study, our findings support the association between OH and not SH with cerebral atrophy, with a more pronounced effect on the anterior-temporal and mediotemporal regions. These results are consistent with the known vulnerability of the mediotemporal lobe and hippocampus to acute and chronic hypoxia due to cerebral hypoperfusion^48^ and suggest that there may be a direct hemodynamic impact of OH on these selected cortical areas.^3,29,49^ Alternatively, the observed atrophy might represent a specific phenotype of patients with OH, characterized by widespread progression of Lewy body pathology. Future research endeavors will be needed to clarify whether an aggressive treatment with vasopressor agents, even at the expense of greater prevalence of SH, may reduce the extent of brain atrophy and result in better short- and long-term outcomes.

## Glossary

BP: blood pressure
CI: confidence interval
DLB: dementia with Lewy bodies
MDS: Movement Disorders Society
MMSE: Mini-Mental State Examination
MoCA: Montreal Cognitive Assessment
OH: orthostatic hypotension
PD: Parkinson disease
SH: supine hypertension
UPDRS: Unified Parkinson's Disease Rating Scale
WMH: white matter hyperintensities
